# Pneumococcal carriage among sickle cell disease patients in Accra, Ghana: Risk factors, serotypes and antibiotic resistance

**DOI:** 10.1371/journal.pone.0206728

**Published:** 2018-11-08

**Authors:** Nicholas T. K. D. Dayie, Georgina Tetteh-Ocloo, Appiah-Korang Labi, Edeghonghon Olayemi, Hans-Christian Slotved, Margaret Lartey, Eric S. Donkor

**Affiliations:** 1 Dept. of Medical Microbiology, School of Biomedical and Allied Health Sciences University of Ghana, Accra, Ghana; 2 Dept. of Microbiology, Korle-Bu Teaching Hospital, Accra, Ghana; 3 Dept. of Haemataology, School of Biomedical and Allied Health Sciences, University of Ghana, Accra, Ghana; 4 Statens Serum Institut, Dept of Microbiological Surveillance and Research, Copenhagen, Denmark; 5 Dept. of Medicine, School of Medicine and Dentistry, University of Ghana, Accra, Ghana; Universidade de Lisboa Faculdade de Medicina, PORTUGAL

## Abstract

**Background:**

Pneumococcal carriage is the precursor for development of pneumococcal disease, and is also responsible for transmission of the organism from person-to-person. Individuals with Sickle Cell Disease (SCD) are more likely to develop invasive disease with *S*. *pneumoniae* compared to their healthy counterparts and the presentation of disease in the former is usually abrupt and severe. In Africa, little is known about the pneumococcus in relation to people with SCD Sickle Cell Disease (SCD). The aim of the study was to investigate the epidemiology of pneumococcal carriage among SCD patients including the carriage prevalence, risk factors, serotypes and antibiotic resistance.

**Method:**

This was a cross sectional study involving 402 SCD patients recruited from Korle Bu Teaching Hospital and Princess Marie Louis Hospital in Accra from October 2016 to March 2017. The study subjects included 202 children of the age groups: ≤5 years (94), >5–9 years (75), ≥10–13 years (33) and 200 adults of the age groups: 14–20 years (46), 21–40 years (112), 41–60 years (25), ≤ 61 years (17). Nasopharyngeal (NP) swabs were collected from the study participants as well as epidemiological data on demographic, household and clinical features. The NP specimens were cultured for *S*. *pneumoniae* and the isolates were serotyped by latex agglutination. Antimicrobial susceptibility tests of the isolates were done by the disc diffusion test and E-test.

**Results:**

Prevalence of *S*. *pneumoniae* carriage among children and adult SCD patients enrolled in the study were 79/202 (39.1%; 95% CI: 32.3 to 46.2) and 20/200 (10.0%; 95% CI: 6.2 to 15.0) respectively. Risk factors associated with pneumococcal carriage were age (OR = 1.137; 95% CI: 1.036–1.248; p = 0.007) and runny nose (OR = 5.371; 95% CI: 1.760–16.390; p = 0.003). Overall, twenty-six pneumococcal serotypes were isolated from the study participants and the predominant serotype was 6B (10.6%), followed by 23B (8.2%). Among the children, serotype coverage of the 13-valent Pneumococcal Conjugate Vaccine, which is currently used in Ghana was 32.4%. Prevalence of penicillin resistance among the pneumococcal isolates was 37.4% (37/99) and all the penicillin-resistant isolates exhibited intermediate penicillin resistance with the exception of one isolate that showed full resistance and was susceptible to ceftriaxone. Prevalence of resistance to the other antibiotics ranged from 2.5% (levofloxacin) to 85% (cotrimoxazole). Multidrug resistance occurred among 34.3% (34/99) of the pneumococcal isolates.

**Conclusion:**

Pneumococcal carriage was four-fold higher in SCD children than adults and was characterized by predominance of non-vaccine serotypes and considerable level of multidrug resistance, though penicillin, cefotaxime and levofloxacin resistance appeared to be very low.

## Background

*Streptococcus pneumoniae* also referred to as pneumococcus, is an encapsulated respiratory pathogen of immense public health significance [1, 2]. An important characteristic of the pneumococcus is that the organism is part of the normal flora of the nasopharynx of humans and carriage of the organism is affected by a wide range of factors such as age, acute respiratory tract infection and immunosuppression [3–5]. Pneumococcal carriage is the precursor for development of pneumococcal disease, and is also responsible for transmission of the organism from person-to-person [6, 7]. Clinically, the pneumococcus causes several invasive and non-invasive diseases including pneumonia, meningitis, septicaemia, sinusitis and acute otitis media. There are about one million new pneumococcal infections every year, majority of which occur in the developing world where children <5 years are most affected [2]. The pneumococcal public health burden is exacerbated by the rising resistance of the organism to several essential antibiotics especially, penicillin, cephalosporins and macrolides [8, 9].

The vast public health burden of the pneumococcus underlies the importance of control through vaccination, and recently, pneumococcal conjugate vaccines (PCVs) are being introduced into the childhood vaccination programmes of many developing countries (www.gavialliance.org, www.view-hub.org). Two types of pneumococcal conjugate vaccines (PCVs) are currently in use and they include the 10-valent vaccine (PCV10) which comprises pneumococcal serotypes 4, 6B, 9V, 14, 18C, 19F, 23F, 1, 5, 7F and the 13-valent vaccine (PCV13) which has three additional serotypes of 3, 6A, 19A [10, 11]. Pneumococcal conjugate vaccines have been shown to be superior to the previous pneumococcal polyvalent polysaccharide vaccine (PPV 23) [6, 7]. Though PPV23 contains 23 serotypes (1, 2, 3, 4, 5, 6B, 7F, 8, 9N, 9V, 10, 11A, 12F, 14, 15B, 17F, 18C, 19A, 19F, 20, 22F, 23F, 33F), it provides limited protection in immune-compromised individuals and infants [10, 11]. Furthermore, a study by Donkor *et al*. [12] showed that pneumococcal serotypes in PPV23 which are not covered by PCVs hardly cause diseases in West Africa. Despite PCVs offering hope in reducing pneumococcal disease burden, they are not a panacea for pneumococcal infections due to the limited serotype composition and possibility of serotype replacement and switching resulting from evolutionary effects of the vaccines [10, 11].

Sickle cell disease (SCD) is a term for a number of genetic disorders in which haemoglobin is structurally abnormal, resulting in the episodic formation of sickle-shaped red blood cells and a wide range of clinical manifestations [13]. It is the most common genetic disorder worldwide, with about 300,000 affected infants born each year [14]. Sickle cell disease patients are prone to developing invasive pneumococcal disease due to the inability of their defective immune system to effectively handle encapsulated bacteria [15]. A study showed that children with SCD are 600 times more likely to develop invasive disease with *S*. *pneumoniae* compared to their healthy counterparts and the presentation of disease in the former is usually abrupt and severe [16]. This led to the establishment of anti-pneumococcal prophylaxis for SCD children including the administration of Penicillin V and PPV23 in the past. The advent of PCVs further provides anti-pneumococcal prophylaxis for SCD children as these vaccines are administered to all children. In Ghana, there is hardly any comprehensive epidemiological data on the pneumococcus in relation to SCD patients, which is a major hindrance to effective control of pneumococcal infections among this at-risk population. To help address these concerns and to provide the necessary information to inform vaccination policies, the current study was carried out. The aim of the study was investigate the epidemiology of pneumococcal carriage among children and adult SCD patients in Ghana, including the prevalence, risk factors, serotypes and antibiotic resistance.

## Methods

### Study site

This research was carried out at the Korle-Bu Teaching Hospital (KBTH) and the Princess Marie Louise Children’s Hospital (PML Children’s hospital) both in the Accra Metropolis. Accra is the capital city of Ghana and has a population of about two million people [17]. The Korle Bu Teaching Hospital is the premier health care facility in Ghana and a major referral health facility in Southern Ghana. The hospital has the Ghana Institute of Clinical Genetics (GICG) which runs a sickle cell disease clinic located on the Korle Bu campus. The clinic runs from Monday to Friday and receives over 10,000 visits per year from patients aged 13 years and older [18]. The PML Children’s hospital is the only children hospital in the Accra Metropolis. It has a sickle cell clinic, which runs once a week with an attendance of about 20 patients [19]. The clinic receives over 800 visits per year from patients below one month to 18 years.

### Study design and sampling

This was a prospective cross -sectional study involving sickle cell disease (SCD) patients at the KBTH and the PML Children’s hospital from October 2016 to March 2017. The study was approved by the Ethical and Protocol Review committees of the College of Health Sciences, University of Ghana and the Ghana Health Service, and written consent was obtained from adult participants and parents or guardians of the minors included in the study. Two hundred (200) sickle cell patients visiting the adult sickle cell clinic at the KBTH and two hundred and two (202) sickle cell patients visiting the PML Children’s Hospital were recruited consecutively in the study. The minimum sample size calculation was based on previous pneumococcal carriage prevalence data in children [5] and adults [20] in Ghana, using a precision of 5% (with 95% confidence) and 90% power. The inclusion criterion for enrollment into the study was SCD patients in a steady state; exclusion criteria were SCD patients who were on admission, or had received blood transfusion in the previous three months, or were on medications apart from those used routinely in the management of the disease. The diagnosis of SCD was based on hemoglobin electrophoresis. A structured questionnaire was used to collected data on risk factors of pneumococcal carriage from the study participants. The questionnaire covered three areas including demographic features, clinical features and household characteristics.

Nasopharyngeal samples were collected from the study participants by a trained nurse according to the WHO recommended procedure for detecting pneumococcal carriage [21] using nylon-tipped paediatric size nasopharyngeal swabs produced by Copan Flock Technologies Srl (Lot L.70VE00). The swab sample was then placed in a cryotube containing 1 ml of skim milk-tryptone-glucose-glycerin (STGG) medium and cut to a length of about 4 cm from the handle of the swab using a pair of scissors disinfected with 70% alcohol. The cryotube containing the cut swab was capped tightly and labeled with a unique ID number and then transported on ice packs within three hours to the Research Laboratory of the Department of Medical Microbiology, School of Biomedical and Allied Health Sciences, University of Ghana. Upon reaching the laboratory, the transport medium containing the swab was vortexed for about 1–2 minutes to disperse the organisms from the tip of the swab into the transport medium and stored at -80°C.

### Specimen analysis

The swab specimens in the transport medium stored at -80°C were brought out and allowed to thaw at room temperature. The thawed specimens were vortexed for about 10–15 seconds to mix thoroughly and uniformly disperse the organisms in the medium. Using a sterile calibrated loop, 10μl volume of the vortexed specimen was transferred from the cryotube and inoculated onto a half portion of two types of 5% sheep blood agar. One of the blood agar plates was made selective for pneumococci by adding gentamicin at a concentration of 5 μg/ml and the other was non-selective on which grew other organisms apart from pneumococci. The inoculated plates were incubated at 37°C with lighted candle overnight (18–20 hours) in a sealed candle jar to generate an atmosphere of 5–10% CO_2_ [22]. After 18–20 hours of incubation, the plates were examined for growth. Alpha-haemolytic colonies were selected for isolation and characterization of *S*. *pneumoniae*. Such colonies were Gram stained and examined for lancet-shaped Gram-positive diplococcic. Serotyping and detection of multiple serotypes of pneumococcal isolates was performed as described by Dayie et al [23]. The pneumococcal isolates were serotyped by the pneumotest latex agglutination kit (SSI Diagnostica, Hillerød, Denmark) and results confirmed by the Quellung reaction using serotype specific antisera (SSI Diagnostica).

Antibiotic susceptibility testing of *S*. *pneumoniae* isolates was done using the Kirby Bauer method, and the antibiotics tested included tetracycline-30 μg, erythromycin-15 μg, cotrimoxazole-25 μg, levofloxacin-5 μg and 1μg oxacillin. Zone diameters formed around the antibiotic discs were measured and classified as sensitive or resistant based on the Clinical Laboratory Standard Institute (CLSI) break point system. Isolates with zone sizes ≤ 19 mm for oxacillin were further tested for penicillin minimum inhibitory concentrations (MIC) using the E-test [22]. Based on CLSI guidelines, isolates with MIC reading ≤ 0.06 μg/ml were considered to be penicillin susceptible; those with MIC’s from 0.12 to 1.0 μg/ml were considered to be intermediate resistant and those ≥ 2 μg/ml were considered to be total penicillin resistance [22]. The MICs for ceftriaxone were determined for all the isolates that showed resistance to penicillin. Isolates with MICs ≤1 were considered to be susceptible to ceftriaxone; those with MICs of 2 μg/ml were considered intermediate resistant and those with MICs of ≥ 4 were considered to be completely resistant [22].

### Data analysis

Data was entered into MS Excel and imported into STATA 11 (Strata Corp, College Station, TX, USA) for analysis. Descriptive analyses including computation of arithmetic means, frequencies and percentages were done on the study variables. The prevalence of nasopharyngeal carriage of *S*. *pneumoniae* was presented as proportions of individuals in different age groups. Univariate associations were performed between pneumococcal carriage and demographic, clinical and household features: analysis of variance was used for numeric variables, whereas chi-square test was used for categorical variables. Logistic regression model was used to analyze exposures associated with carriage and the results were presented as Odds Ratios (OR), p values and Confidence Intervals (95% CI). Serotype distribution was evaluated and impact of pneumococcal vaccination among the SCD children and adults was estimated by the theoretical coverage of PCV10, PCV13 and PPV23. Antibiogram and multidrug resistance of pneumococcal isolates were computed; multidrug resistance was defined as resistance to penicillin and two or more classes of antimicrobial agents.

## Results

### Demographic, household and clinical features of the study participants

The 402 SCD patients enrolled in the study were made up of 202 children with ages ranging from 1 to 13 years (mean age: 3.4 ± 1.9) and 200 adults with ages ranging from 14 to 82 years (mean age: 32.4 ± 15.1). The children comprised of 111 males and 91 females, while the adults were made up of 76 males and 124 females. Overall, 86.3% (347) of the study participants were Christians, 57.2% (230) lived in compound houses, and none was exposed to passive smoking. A large majority of 93.6% (189) of the children were attending school. A proportion of 47% (95) of the children and 61.5% (123) of the adults had respiratory symptoms, and runny nose was the most common respiratory symptom in both populations. Asthma, Pneumonia and Otitis Media occurred in both children and adult populations: Otitis Media was slightly more common in children (1.5%, n = 3) than adults (1%. n = 2), while Asthma and Pneumonia were relatively common in Adults; Asthma occurred in 6.5% (13) and 3.5% (7) of adults and children respectively, while Pneumonia occurred in 8.5% (17) of the adults and 6.9% (14) of the children. A proportion of 3.5% (7) of the SCD children were on penicillin V prophylaxis, while 44.6% (90) had received PCV13 (3 doses), the large majority (84, 93.3%) of whom were under five years. The demographic, household and clinical features of the study participants are summarised in Table 1.

**Table 1:** Demographic, household and clinical features of the study participants.

Characteristics	Children		Adults	
	N	%	N	%
**Gender**				
*Male*	111	55	76	38.0
*Female*	91	45	124	62.0
**School attendance**	189	93.6	N/A	N/A
**Religion**				
*Christianity*	170	84.2	177	88.5
*Islam*	32	15.8	23	11.5
**Type of residence**				
*Compound house*	129	63.9	101	50.5
*Self-contained*	73	36.1	99	49.5
**Number of persons per house**				
*< 5*	71	35.1	78	39.0
*5–10*	118	58.4	102	51.0
*11–20*	12	5.9	20	10.0
*≥ 21*	1	0.5	0	0
**Exposure to passive smoking**	0	0.0	0	0.0
**Asthma**	7	3.5	13	6.5
**Pneumonia**	14	6.9	17	8.5
**Respiratory symptoms**				
*Sore throat*	18	8.9	40	20.0
*Chest pains*	16	7.9	71	35.5
*Runny nose*	62	30.7	79	39.5
*Blocked nose*	48	23.8	59	29.5
*Productive cough*	41	20.3	43	21.5
*Difficulty in breathing*	10	5.0	26	13.0
**Ear Infection**	3	1.5	2	1.0

N- number; Mean ages of children and adults were 3.4 ± 1.90 and 32.4 ± 15.1 respectively; Total number of children and adults were 202 and 200 respectively.

### Pneumococcal carriage and risk factors

Prevalence of *S*. *pneumoniae* carriage among children and adult SCD patients enrolled in the study were 79/202 (39.1%; 95% CI: 32.3 to 46.2) and 20/200 (10.0%; 95% CI: 6.2 to 15.0) respectively. Carriage among different children age groups of ≤5 years, >5–9 years and ≥10–13 years were, 47.9% (45/94), 34.7% (26/75) and 24.2% (8/33) respectively. Carriage among different adult age groups of 14–20 years, 21–40 years, 41–60 years and ≤ 61 years were 6.5% (3/46), 9.8% (11/112), 20% (5/25) and 5.9% (1/17) respectively.

The multivariate analysis showed that pneumococcal carriage was significantly associated with age (OR = 1.137; 95% CI: 1.036–1.248; p = 0.007) and runny nose (OR = 5.371; 95% CI: 1.760–16.390; p = 0.003) among the children. However, among the adults, pneumococcal carriage was not significantly associated with any of the demographic, household and clinical variables investigated.

### Pneumococcal carriage serotypes

**Fig 1:**
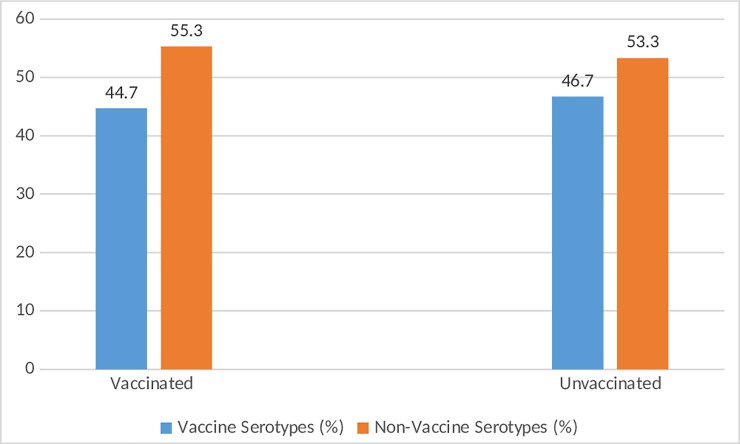
Distribution of vaccine and non-vaccine *S*. *pneumoniae* serotypes between vaccinated and unvaccinated children with SCD. Non-vaccine serotypes were relatively more common in both vaccinated and unvaccinated children; there was no significant association between vaccination status and the distribution of vaccine and non-vaccine serotypes at *p*<0.05.

Serotyping was performed on 85 of the 99 pneumococci isolated from the study participants as 14 isolates had lost viability. Overall, 26 pneumococcal serotypes were identified and the predominant serotype was 6B (10.6%), followed by 23B (8.2%), 32F (7.1%) and 11A (7.1%). The distribution of serotypes in children and adult SCD patients and their coverage by various pneumococcal vaccines are shown in Table 2. In the adult population, eleven different serotypes were carried and generally, these serotypes appeared to be evenly distributed. In the children population, twenty-two different serotypes were carried and the most common was serotype 6B (%). There are several serotypes that appeared to be commonly carried by children that were not carried by adults; these serotypes were 23B, 32F and 19F. Overall, serotype coverage of PCV10, PCV13 and PPV23 were 27.1%, 29.4% and 49.4% respectively (not shown in table). Among the children SCD population, serotype coverage by the various vaccines were PCV10 (29.6%), PCV13 (32.4%) and PPV23 (47.9%), while among the adult population, the coverage was PCV10 (14.3%), PCV13 (14.3%) and PPV23 (57.1%). The distribution of vaccine and non-vaccine serotypes of pneumococcus among vaccinated and unvaccinated children is illustrated in Fig 1, which shows that there was no significant association between vaccination status and distribution of the two categories of serotypes.

**Table 2:** *S*. *pneumoniae* serotypes isolated from SCD patients.

Serotype	Overall N (%)	Children N (%)	Adult N (%)	Serotype included in vaccine
3	5 (5.9)	4 (5.6)	1 (7.1)	PCV13, PPV23
6A	2 (2.4)	2 (2.8)	0 (0)	PCV13
6B	9 (10.1)	8 (11.3)	1 (7.1)	PCV10, PCV13, PPV23
7F	2 (2.4)	1 (1.4)	1 (7.1)	PCV10, PCV13, PPV23
8	2 (2.4)	1 (1.4)	1 (7.1)	PPV23
9A	1 (1.2)	1 (1.4)	0 (0)	Non-vaccine serotype
10A	5 (5.9)	3 (4.2)	2 (14.3)	PPV-23
11A	6 (7.1)	5 (7.0)	1 (7.1)	PPV-23
11D	1 (1.2)	1 (1.4)	0 (0)	Non-vaccine serotype
13	1 (1.2)	1 (1.4)	0 (0)	Non-vaccine serotype
14	3 (3.5)	3 (4.2)	0 (0)	PCV10, PCV13, PPV23
15A	3 (3.5)	3 (4.2)	0 (0)	Non-vaccine serotype
15C	4 (4.7)	2 (2.8)	2 (14.3)	Non-vaccine serotype
16F	2 (2.4)	2 (2.8)	0 (0)	Non-vaccine serotype
17A	1 (1.2)	1 (1.4)	0 (0)	Non-vaccine serotype
17F	1 (1.2)	0 (0)	1 (7.1)	PPV23
19B	1 (1.2)	1 (1.4)	0 (0)	Non-vaccine serotype
19F	5 (5.9)	5 (7.0)	0 (0)	PCV10, PCV13, PPV23
21	1 (1.2)	1 (1.4)	0 (0)	Non-vaccine serotype
23B	7 (8.2)	7 (9.9)	0 (0)	Non-vaccine serotype
23F	4 (4.7)	4 (5.6)	0 (0)	PCV10, PCV13, PPV23
28F	1 (1.2)	0 (0)	1 (7.1)	Non-vaccine serotype
31	1 (1.2)	1 (1.4)	0 (0)	Non-vaccine serotype
32F	6 (7.1)	6 (8.5)	0 (0)	Non-vaccine serotype
34	5 (5.9)	4 (5.6)	1 (7.1)	Non-vaccine serotype
38	1 (1.2)	0 (0)	1 (7.1)	Non-vaccine serotype
Non-typeable	5 (5.9)	4 (5.6)	1 (7.1)	Not applicable

N- number; Total number of serotypes for children, adults and overall were 71, 14 and 85 respectively

### Pneumococcal antibiotic resistance

Among the 79 pneumococcal isolates from children SCD patients, prevalence of resistance to penicillin (oxacillin), levofloxacin, erythromycin, tetracycline and cotrimoxazole were 36.7% (29), 2.5% (2), 15.2% (12), 70.9% (56), 75.9% (60) respectively; in the case of adult SCD patients, prevalence of resistance among the 20 pneumococci isolated were 40% (8), 10% (2), 10% (2), 80% (16), 85% (17) for penicillin (oxacillin), levofloxacin erythromycin, tetracycline and cotrimoxazole respectively. More detailed analysis of penicillin resistance of the pneumococcal isolates is shown in Table 3. Overall, 37 (37.4%) of the 99 pneumococcal isolates from the study subjects were resistant to penicillin. With the exception of one isolate (from a one year old child) that showed full resistance to penicillin with MIC of 2 μg/ml, all the penicillin-resistant pneumococci exhibited intermediate resistance (36.4%, 36/99). Among the children, the 10–13 years group had the highest prevalence of penicillin-resistant pneumococci, while among the adults, the highest prevalence occurred among the 14–20 years group (66.7%). All the penicillin-resistant isolates tested against ceftriaxone using the E-test were susceptible to the drug. Multidrug resistance occurred in 34.3% (34) of the pneumococcal isolates from the study participants; prevalence of multidrug resistance in children and adult SCD patients were 32.9% (26) and 40% (8) respectively. The commonest pattern of multidrug resistance among the pneumococcal isolates was resistance to penicillin (intermediate resistance), tetracycline and cotrimoxazole.

**Table 3:** Carriage of penicillin-resistant *S*. *pneumoniae* among SCD patients.

	Age	Carriage N (%)	Intermediate Resistance (MIC = 0.12–1.0 μg/ml) N (%)	Full Resistance (MIC = ≥ 2.0 μg/ml) N (%)	PRSP N (%)
**Children**	**≤ 5 yrs**	45 (47.9)	15 (33.3)	1 (2.2)	16 (35.6)
	**>5–9 yrs**	26 (34.7)	9 (34.6)	0 (0)	9 (34.6)
	**10–13 yrs**	8 (24.2)	4 (50.0)	0 (0)	4 (50.0)
**Adults**	**14–20 yrs**	3 (6.5)	2 (66.7)	0 (0)	2 (66.7)
	**21–40 yrs**	11 (9.8)	4 (36.4)	0 (0)	4 (36.4)
	**41–60 yrs**	5 (20)	2 (40.0)	0 (0)	2 (40.0)
	**≥ 61 yrs**	1 (5.9)	0 (0.0)	0 (0)	0 (0.0)

## Discussion

This study is one of the very few to report on pneumococcal carriage among SCD patients in Ghana, and it is novel as the other studies did not report on pneumococcal serotypes, which is crucial for evaluating the current pneumococcal vaccines in this at-risk population. We observed an overall carriage prevalence of 39.1% and 10% in children (≤13 years) and adults (>13 years) respectively. By comparison, Donkor *et al*. [24] reported a much lower prevalence of 10% among SCD children ≤13 years in the same study area. A similar study by Baffoe-Bonnie *et al*. [25] among SCD children ≤5 years in Kumasi (a city in Ghana) reported a pneumococcal carriage prevalence of 16%, which is much lower than the prevalence (47.9%) observed among the same age group in the current study. Pneumococcal carriage prevalence among children with SCD reported in other countries were 33% in Uganda [26], 14% in Gabon [27], 13% in the USA [28] and 21% in the UK [29]. In Ghana, the relatively higher prevalence of pneumococcal carriage observed among SCD children in this study could be partly due to the small proportion of 7% who were on penicillin prophylaxis compared to over 50% in the study of Donkor *et al*. [24] and Baffoe-Bonnie *et al*. [25]. Several lines of evidence indicate that penicillin prophylaxis significantly reduces pneumococcal carriage in SCD patients [26, 30], which could not be investigated in this study due to insufficient data on penicillin prophylaxis. The relatively small proportion of SCD children who received penicillin prophylaxis in the current study could be due to the availability and routine use of pneumococcal vaccines in children in Ghana compared to the pre-vaccination period when the studies by Donkor *et al*. [24] and Baffoe-Bonnie *et al*. [25] were carried out.

The risk factors (runny nose and age) of pneumococcal carriage observed among children in this study have been reported by other studies in Ghana and elsewhere, though not among SCD patients [5, 31–37]. Carriage of pneumococcus has been reported to be particularly high among children less than five years; the colonization rate rises from birth until it peaks around the age of 1–2 years, and thereafter an age related decline is observed [4, 38]. The association of respiratory symptoms (runny nose) with carriage of pneumococcus in this study may be due to the damage respiratory symptoms cause to the respiratory tract, which increases the chance of acquiring pneumococcus [6, 7]. Several risk factors of pneumococcal carriage reported by other investigators, such as school attendance (daycare centres/kindergarten) and history of acute asthma [5, 39] were not observed in our study. This partly shows that pneumococcal carriage risk factors may vary from one population to another, though some risk factors may be relatively more common across different populations. It could also be that our sample was not well suited to evaluate the effects of some of these risk factors. For example, the children in our sample had a wider age range and almost all of them attended school, which limits any analysis to evaluate the effect of daycare centres/kindergarten attendance on pneumococcal carriage.

Penicillin has been a very important antibiotic in the treatment of pneumococcal infections, and in the past two to three decades, penicillin-resistant pneumococci have become a global problem. In this study, full penicillin resistance was rare occurring in only one pneumococcal isolate, an observation which concurs with the study by Donkor *et al*. [24] who did not observe any penicillin resistant isolates carried by SCD children in Ghana. It is important to note that the study by Donkor *et al*. [24] categorized pneumococcal isolates as sensitive or resistant without reporting on intermediate resistance. The pneumococcal carriage study by Baffoe-Bonnie *et al*. [25] among SCD children in Ghana, however did report on pneumococcal intermediate and full penicillin; intermediate resistance was 44% while there were no full resistant isolates. In a pneumococcal carriage study of healthy children less than six years in Ghana, Dayie *et al*. [23] reported that 45% of the isolates showed intermediate penicillin resistance while two isolates were fully penicillin resistant. These observations confirm the rare occurrence of full penicillin-resistant pneumococci observed in our data as well as the significant magnitude of intermediate penicillin resistance of 36.4%. The high prevalence of pneumococcal resistance to tetracycline and cotrimoxazole has been previously reported in Ghana not only for pneumococcus but several other bacterial pathogens [40–45], and may be directly linked to usage of these drugs. In Ghana, tetracycline and cotrimoxazole have been on the market for a very long time; this, coupled with the high rates of self-medication in the country [46], contributes significantly to the high pneumococcal resistance observed in this study. The relatively lower prevalence of resistance observed for cefotaxime, levofloxacin and erythromycin in this study is probably because these antibiotics have been on the market for a relatively short period, and may not have been subjected to high usage like cotrimoxazole and tetracycline. In addition, some of these drugs such as cefotaxime are expensive and this tends to limit their usage in Ghana where the incomes of many people are still low. Apart from antibiotic use, pneumococcal resistance tends to be associated with specific serotypes and clones of the organism [47–49]. Owing to the limited numbers of the different pneumococcal serotypes in our data, it was difficult to perform any meaningful analysis to evaluate this relationship. It is however, of interest to note that the only full penicillin resistant isolate in this study was identified as serotype 19F, which has been previously associated with antibiotic resistance [50]. The prevalence of pneumococcal multidrug resistance observed among both adult and children SCD populations (>30%), concurs with the increasingly high pneumococcal multidrug resistance of 7.8–87%% observed in Ghana in the last decade [5, 24, 51]. This is very worrying in the case of SCD patients who have a relatively higher risk of pneumococcal infections. The current situation of pneumococcal multidrug resistance in Ghana highlights the need for surveillance of antibiotic resistance of this important pathogen in the country to ensure successful treatment of its infections.

The predominant pneumococcal serotypes in this study (serotypes 6B, 23B, 32F, 11A) were all reported in a post-vaccination carriage survey among healthy children in Ghana [52]. While there is no pre-vaccination pneumococcal serotype data on SCD patients in Ghana, a pre-vaccination carriage survey among healthy children in the country reported four predominant pneumococcal serotypes including 19F, 6B, 6A and 23F [23]. It is important to interpret the serotype data of this study in the light of the introduction of PCV13 in Ghana considering the significance of the vaccine to SCD patients. Ghana initiated routine PCV13 immunisation in 2012 and the vaccine is given to all infants at 6, 10, 14 weeks. The current vaccination coverage of PCV13 in Ghana is over 80% [53], though in our sample 44.6% had received all three doses of the vaccine. The low serotype coverage of PCV13 (29.4%) in the current study is due to two main non-vaccine serotypes, 23B and 32F that were among the common serotypes carried by the study subjects particularly, children. These two serotypes especially, 32F had been rarely reported in Ghana in the pre-vaccination period [5, 23]. Consequently, their relatively common occurrence in carriage could indicate serotype replacement as a result of introduction of PCV13 in Ghana, though further surveillance data is needed to confirm this. Serotype replacement carriage of pneumococcal vaccine serotypes with non-vaccine types is well known and to be expected in populations immunised with PCVs [54–56]. It is important to note that, in the post-vaccination carriage survey among healthy children in Ghana mentioned above, serotype 23B was the most prevalent serotype reported [52]. In the case of serotype 32F, our data appears to be the first report on serotype replacement globally. The similar prevalence of vaccine serotypes or non-vaccine serotypes in vaccinated and non-vaccinated SCD children probably indicate herd immunity associated with the wide coverage of PCV13 in Ghana, which has been reported elsewhere [57, 58]. Competition between vaccine and non-vaccine serotypes plays a vital role in the herd immunity observed in the post-vaccination era of PCVs [59]. By reducing carriage colonization of vaccine serotype carriage, PCVs provide non-vaccine serotypes a competitive advantage over vaccine types in the nasopharynx. Thus, in vaccinated populations, colonization of vaccine serotypes in vaccinated individuals is suppressed resulting in reduced transmission to unvaccinated contacts. Furthermore, at the population level, there is competition between vaccine serotypes and non-vaccine serotypes in non-vaccinated individuals, and also the spread of vaccine serotypes is likely inhibited by competition from non-vaccine types in non-vaccinated people [59]. This phenomenon of serotype replacement also accounts for the non-significant association between pneumococcal carriage prevalence and vaccination with PCV13 in this study, which also been reported in other studies [60, 61]. In view of the low serotype coverage and potential evolutionary problems of PCV13 observed in this study, penicillin prophylaxis among SCD patients in Ghana should be encouraged in this era of pneumococcal conjugate vaccines. In connection with this, there is the need for further studies to see how these carriage dynamics affects invasive pneumococcal disease among SCD individuals.

The study concludes that, the pneumococcus is commonly carried among SCD children in Accra particularly, those *≤* 5 years and the main carriage risk factors are age and runny nose. There is a low serotype coverage of PCV13 among SCD children and adult populations in Accra. This highlights the need for maximizing effective antibiotic treatment and prophylaxis among the study population in the case that the replacing non-vaccine serotypes are causing disease. Pneumococci carried by the SCD patients hardly showed full penicillin resistance, providing evidence for the use of this antibiotic among SCD patients against pneumococcal infections. In addition, cefotaxime and levofloxacin are suitable for treating pneumococcal infections among the SCD patients.
