# Correction: Pneumococcal carriage among sickle cell disease patients in Accra, Ghana: Risk factors, serotypes and antibiotic resistance

**DOI:** 10.1371/journal.pone.0211838

**Published:** 2019-01-30

**Authors:** The PLOS ONE Staff

There is an error in the article XML that causes the PubMed citation to be indexed incorrectly. The publisher apologizes for the error.

## References

[1] Dayie NTKD, Tetteh-Ocloo G, Labi A-K, Olayemi E, Slotved H-C, Lartey M, et al (2018) Pneumococcal carriage among sickle cell disease patients in Accra, Ghana: Risk factors, serotypes and antibiotic resistance. PLoS ONE 13(11): e0206728 10.1371/journal.pone.0206728 30408061 PMC6224078

